# Research on the correlation between clinical nurses’ self-efficacy, future time perspective, and occupational burnout

**DOI:** 10.3389/fpubh.2024.1363450

**Published:** 2024-06-17

**Authors:** Daixun Xie, Xiaoqin Zhu, Xiurong Zhang, Zhaoquan Jiang, Xin Wang, Tao Liu

**Affiliations:** ^1^Department of Nursing, Jinzhou Medical University, Jinzhou, China; ^2^Emergency Department, Yibin Second People's Hospital, Yibin, China; ^3^Department of Nursing, Huaian Hospital of Huaian City, Huaian, China

**Keywords:** future time perspective, job burnout, self-efficacy, mediating role, clinical nurses

## Abstract

**Background:**

The number of clinical nurses in China experiencing professional burnout is increasing yearly, posing a serious challenge to the public health sector. Implementing effective intervention strategies is key to reducing the level of occupational burnout. At present, training aimed at alleviating occupational burnout among clinical nurses is very limited, with common training programs focusing on addressing external factors of occupational burnout rather than the internal cognitive issues of clinical nurses. Self-efficacy and future time perspective are both aspects of an individual’s internal self-cognition. Meanwhile, the relationship between clinical nurses’ self-efficacy, future time perspective, and occupational burnout is not clear, and further research is needed to verify this.

**Objective:**

This study aims to reveal the relationship between clinical nurses’ self-efficacy, future time perspective, and occupational burnout, and to explore the mediating role of future time perspective between self-efficacy and occupational burnout among clinical nurses, providing a scientific reference for training directions to improve occupational burnout.

**Methods:**

This study used a cross-sectional design, conducting a questionnaire survey with 529 practicing clinical nurses using the General Demographics Questionnaire (GDQ), the General Self-Efficacy Scale (GSES), the Zimbardo Time Perspective Inventory (ZTPI), and the Maslach Burnout Inventory-General Survey (MBI-GS). SPSS software version 26.0 was used to analyze the correlation between variables, and AMOS 26.0 was used to test the mediation effect.

**Results:**

Clinical nurses’ self-efficacy had a negative predictive effect on occupational burnout (r = −0.503, *p* < 0.001). Future time perspective showed significant differences in regression coefficients on both the paths of self-efficacy (r = 0.615, *p* < 0.001) and occupational burnout (r = −0.374, *p* < 0.001). Future time perspective played a partial mediating role between self-efficacy and occupational burnout, accounting for 33.8% of the total effect.

**Conclusion:**

This study suggests a significant correlation between clinical nurses’ self-efficacy, future time perspective, and occupational burnout. Self-efficacy can directly affect occupational burnout in clinical nurses and can also indirectly affect occupational burnout through the future time perspective.

## 1 Introduction

Occupational burnout is a state of physical and mental exhaustion caused by occupational stress. It was first proposed by Freudenberger (1), who saw it as a symptom of emotional depletion, particularly prevalent in the service industry. Occupational burnout comprises three dimensions: emotional exhaustion, depersonalization, and reduced personal accomplishment. Emotional exhaustion is the core dimension, manifesting as individuals gradually resisting work under stress, losing enthusiasm, and finding it difficult to engage fully in their jobs. Depersonalization reflects the dimension of interpersonal interaction; it refers to an individual’s adoption of a negative attitude when facing work, consciously maintaining distance from work partners. Reduced personal accomplishment reflects the self-evaluation dimension, indicating that individuals tend to negatively evaluate themselves and experience a decline in work capability and achievement. These three dimensions mutually influence and promote each other (2–5). “In the ‘Summary of General Secretary Xi Jinping’s Important Expositions on Healthy China,’ it is explained: Nursing personnel are an essential force in safeguarding human health and preventing the threat of diseases, and their role should not be overlooked (6). This demonstrates that the development of the nursing profession is increasingly being valued at the social service demand level. However, medical and nursing staff are among the groups most prone to occupational burnout (7). Existing research indicates that domestic nurses experience moderate to high levels of occupational burnout, with a detection rate of 69.21% (8). The incidence of occupational burnout in nurses at China’s top-tier hospitals ranges from 55.4% to 70.05% (9). Once clinical nurses experience occupational burnout, if it is not identified and adjusted in time, various physical illnesses can be triggered, commonly including symptoms such as headaches, insomnia, and gastrointestinal disturbances. Prolonged occupational burnout, if unresolved, not only threatens the physical and mental health of nurses but can also affect their work attitude and engagement. Ultimately, it may impact the quality of nursing services and result in consequences such as talent attrition (10–12).

Self-efficacy is the core concept of the social cognitive theory by American Psychologist Bandura. It refers to an individual’s confidence or belief in their capability to achieve behavioral goals in a specific domain (13). Nurse self-efficacy is the subjective judgment of nurses regarding their ability to competently perform nursing tasks. As a positive factor, self-efficacy plays a role in controlling and regulating behavior and is important in enhancing individual proactivity, adaptability, emotional experiences, and behavioral outcomes. However, when this positive factor fails to achieve the expected results, it can lead to feelings of burnout. Studies show that a strong sense of self-efficacy can enable clinical nurses to better fulfill their responsibilities, be confident in achieving career development goals, and reduce occupational burnout (14). Future time perspective is a personality trait that encompasses emotional, cognitive, and behavioral aspects, representing an individual’s self-awareness of their developmental potential. It can influence an individual’s present behavior and activities (15). People who often indulge in envisioning the future are more likely to adapt to their current professional environment (16). Self-efficacy can affect the coping abilities of newly hired nurses when faced with stress, thereby helping them to better grasp future development trends. Additionally, a positive future time perspective can encourage clinical nurses to set personal future goals and implement them according to plan, enhancing their sense of professional fulfillmentt (17–21).

The Job Demands-Resources model (JD-R) is a significant theory in the field of positive psychology applied to human resource management (22, 23). This model suggests a notable correlation between individual resources and occupational burnout, where the psychological aspects of individual resources can impact occupational burnout. Self-efficacy is a type of personal resource, and bolstering this resource can help guide nurses to view the future with the right attitude, alleviate negative emotions caused by job stress, and thus diminish the negative effects. Past studies have shown that self-efficacy can modulate and mitigate occupational burnout in teachers and predict their job performance, thereby enhancing work adaptability and career exploration attitudes (24–26). Self-efficacy can positively predict future time perspective, aiding individuals in better understanding future time arrangements and, as a result, reducing the incidence of depression. At the same time, future time perspective has a negative predictive effect on teachers’ occupational burnout. However, there is currently a lack of research examining the relationship between self-efficacy, future time perspective, and occupational burnout simultaneously. Moreover, research in China on future time perspective predominantly revolves around the teaching profession, with very few studies focusing on the future time perspective of clinical nurses (27, 28). Therefore, the following hypothesis is proposed: there is a significant correlation between clinical nurses’ self-efficacy, future time perspective, and occupational burnout, and future time perspective may serve as a mediator between self-efficacy and occupational burnout (29–34).

In summary, this study aims to explore the relationship between clinical nurses’ self-efficacy, future time perspective, and occupational burnout, as well as the mediating role of future time perspective between self-efficacy and occupational burnout. This will enrich the body of research on future time perspective and investigate whether it is a predictive factor for the level of occupational burnout among clinical nurses. The goal is for nursing managers to take effective measures from the perspectives of self-efficacy and future time perspective to reduce the level of occupational burnout among clinical nurses, stabilize the nursing workforce, and improve the quality of nursing services.

## 2 Objects and methods

### 2.1 Participants and procedures

The survey was conducted using a questionnaire designed with Questionnaire Star. We contacted the nursing department of hospitals to obtain approval for the research principles and objectives. In October 2023, the questionnaire was administered under the guidance of head nurses in various departments who instructed staff to fill it out diligently. Inclusion criteria included: (1) registered nurses with a valid nursing license; (2) nurses with ≥1 year of nursing work experience and the capability to perform independent nursing duties; (3) informed consent and willing participation in the study. Exclusion criteria included: (1) nurses who were not on duty for ≥6 months during the survey period due to illness, maternity leave, or other reasons; (2) nurses who had been administratively punished by the hospital or administrative authorities for various reasons. A total of 568 questionnaires were collected, with 39 excluded due to responses taking less than 60 s or featuring 10 consecutive identical answers. In the end, 529 valid questionnaires were received, representing a 93.13% effective response rate.

### 2.2 Ethical approval and participant consent

The Ethics Committee of Jinzhou Medical University approved this study (JZMULL2023152). Before the research commenced, participants received written information about the purpose and procedures of the study and completed an informed consent form.

### 2.3 Research instruments

#### 2.3.1 General demographic questionnaire

Based on relevant literature, researchers designed a general information questionnaire to survey the demographic information of clinical nurses, including gender, education level, age, marital status, years of work, and professional title.

#### 2.3.2 Maslach burnout inventory

The Maslach burnout inventory (MBI-GS), developed by Maslach and Jackson (35) and later revised for all professions, was adapted into a Chinese version suitable for the country’s context by Li Chaoping et al. This instrument consists of 15 items across three dimensions: emotional exhaustion (5 items), depersonalization (4 items), and reduced personal accomplishment (6 items) (36). It uses a 7-point Likert scale ranging from 0 (never) to 6 (very frequently). Emotional exhaustion and depersonalization are scored positively, with higher scores indicating a higher level of burnout, while reduced personal accomplishment is scored negatively, with lower scores indicating a higher level of burnout. The total score ranges from 0–90, with <45 indicating a low level, 45–75 a moderate level, and >75 a high level of burnout. In this study, the questionnaire’s Cronbach’s alpha was 0.919, with the three dimensions having Cronbach’s alpha values of 0.957, 0.813, and 0.941, respectively. The Kaiser-Meyer-Olkin (KMO) value was 0.931, and the Bartlett’s Test of Sphericity yielded a chi-square value of 9271.187 (*p* < 0.001), indicating strong reliability and validity of the scale.

#### 2.3.3 General self-efficacy scale

The General Self-Efficacy Scale (GSES), developed by Schwarzer (37) and adapted into Chinese by Wang Caikang (38), is a unidimensional scale consisting of 10 items. It includes statements such as “Whatever happens, I can manage.” A 4-point Likert scale is used, from 1 (not at all true) to 4 (exactly true), with total scores ranging from 10 to 40 points. Higher scores indicate greater self-efficacy: 10–19 is considered low, 20–29 medium, and 30–40 high. In this study, the scale’s Cronbach’s alpha was 0.933, the KMO value was 0.942, and the Bartlett’s Test of Sphericity yielded a chi-square value of 3,930.728 (*p* < 0.001), demonstrating good reliability and validity.

#### 2.3.4 Zimbardo time perspective inventory

Zimbardo and Boyd (39) developed the ZTPI, which consists of 56 items across five subscales. This study focused on the future dimension, as it reflects the orientation toward achieving future goals and is characterized by planning, with a total of 13 items including “I complete tasks methodically and on time” and “I make a list of things to do.” Using a 5-point Likert scale ranging from 1 (not at all true) to 5 (exactly true), the total score ranges from 13 to 65 points. Higher scores indicate a stronger insight into the future development of one’s career. In this study, the Cronbach’s alpha for the questionnaire was 0.895, the KMO value was 0.927, and the Bartlett’s Test of Sphericity yielded a chi-square value of 3406.925 (*p* < 0.001), indicating good reliability and validity.

### 2.4 Statistical methods

Data were analyzed using SPSS 26.0 and AMOS 26.0 software for statistical analysis and structural equation modeling. Demographic data were presented as frequencies and percentages (%), while scores for occupational burnout, time perspective, and self-efficacy were expressed as means ± standard deviations (Mean ± SD). Pearson correlation analysis was used to examine relationships between variables, and the significance of the mediation model was tested with the Bootstrap method. The significance level was set at α = 0.05, with *p* < 0.05 indicating statistically significant differences.

## 3 Results

### 3.1 General information and burnout status of study participants

A total of 529 clinical nurses participated in the study, including 493 female nurses (93.2%), 344 nurses aged 20–30 years (65.0%), 280 unmarried nurses (52.9%), 302 nurses with a bachelor’s degree (57.1%), 284 nurses with 1–5 years of work experience (53.8%), and 280 nurses with moderate levels of occupational burnout (52.9%). For more details, see Table 1.

**Table 1:** The demographics of participants (*n* = 529).

Factors	Group	*N*	%
Gender	Male	36	6.8
	Female	493	93.2
Age (years)	20–30	344	65.0
	31–40	139	26.3
	41–50	33	6.2
	>50	13	2.5
Marriage	Unmarried	280	52.9
	Married	241	45.6
	Divorce	8	1.5
Education	Junior college	217	41.0
	Undergraduate	302	57.1
	postgraduates	10	1.9
Working years	1–5	284	53.8
	5–10	83	15.7
	>10	162	30.6
Professional title	Nurse	258	48.8
	Primary nurse	128	24.2
	Nurse-in-charge	112	21.2
	Deputy chief nurse	26	4.9
	Chief Nurse	5	0.9
Burnout status	Gently	224	42.3
	Moderate	280	52.9
	Severe	25	4.7

### 3.2 Scores on self-efficacy, future time perspective, and occupational burnout among clinical nurses

The average score for occupational burnout was 49.15 ± 16.35, for self-efficacy it was 25.97 ± 6.20, and the score for future time perspective was 43.46 ± 10.29. For more details, see Table 2.

**Table 2:** Total scores on self-efficacy, future time perspective, and occupational burnout scales (*n* = 529).

Variable	Min	Max	Mean ± SD
Job burnout	17	90	49.15 ± 16.35
Self-efficacy	10	40	25.97 ± 6.20
Future time perspective	14	65	43.46 ± 10.29

### 3.3 Correlation analysis of clinical nurses’ self-efficacy, future time perspective and occupational burnout

There was a positive correlation between self-efficacy and future time perspective (r = 0.615, *p* < 0.001), a negative correlation between occupational burnout and self-efficacy (r = −0.503, *p* < 0.001), and a negative correlation between occupational burnout and future time perspective (r = −0.374, *p* < 0.001). For more details, see Table 3.

**Table 3:** Correlation analysis of clinical nurses’ self-efficacy, future time perspective, and occupational burnout (*n* = 529).

Variable	Self-efficacy	Future time perspective	Job burnout
Self-efficacy	1		
Future time perspective	0.615^**^	1	
Job BURNOUT	−0.503^**^	−0.374^**^	1

**p < 0.01.

### 3.4 Mediating structural equation modeling

**Figure 1:**
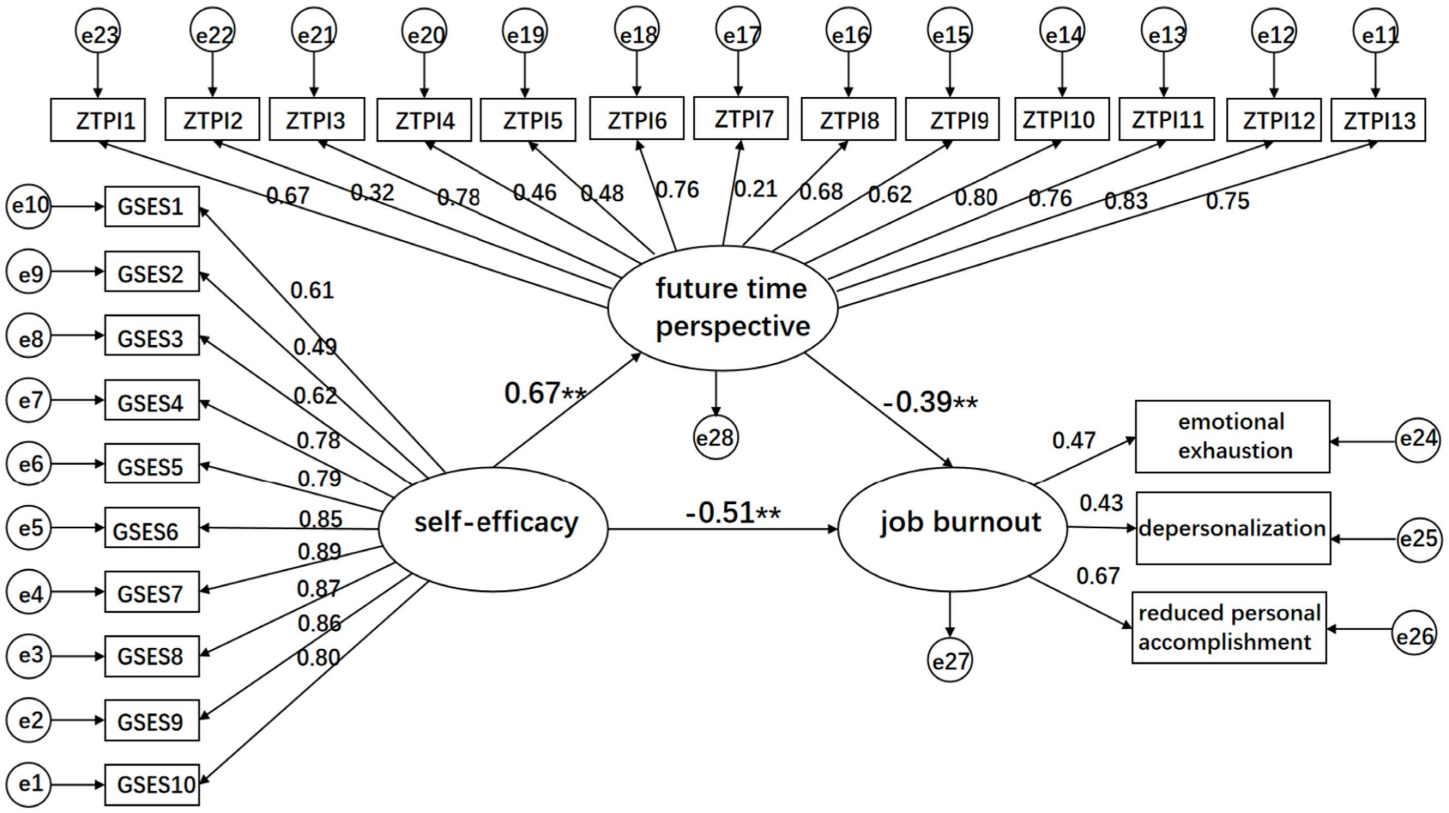
Structural equation modeling of future time perspective as a mediator between self-efficacy and occupational burnout.

Parameters were estimated using the maximum likelihood estimation method, and the model fit was evaluated based on fit indices. The model structure was then tested and modified according to modification indices, resulting in a path diagram that describes the relationships between self-efficacy, future time perspective, and occupational burnout (Figure 1). The model fit met the ideal standards and showed good adaptability. See Table 4 for more details.

**Table 4:** Structural equation model fitting index (*n* = 529).

Project	χ^**2**^	χ^**2**^/df	GFI	AGFI	NFI	IFI	TLI	CFI	RMSEA
Fitting index	770.370	2.970	0.891	0.852	0.914	0.941	0.926	0.941	0.061
Acceptable standards	-	**<**3	**>**0.8	**>**0.8	**>**0.9	**>**0.9	**>**0.9	**>**0.9	**<**0.08

### 3.5 Mediation effect of future time perspective on self-efficacy and occupational burnout

The significance of the mediation effect was tested using the Bootstrap method with 2,000 resamples. The standardized estimate of the indirect effect was −0.262, with a confidence interval that did not include 0, indicating a significant mediation effect. This suggests that future time perspective plays a partial mediating role in the relationship between self-efficacy and occupational burnout, accounting for 33.8% of the total effect. For more details, see Table 5.

**Table 5:** Decomposition table of total effect, direct effect, and mediation effect (*n* = 529).

Project	Path coefficient	SE	95% confidence interval	*P*-value	Relative effect value
Direct effect	−0.512	0.102	[−0.716,-0.314]	*p* < 0.01	66.2%
Indirect effect	−0.262	0.074	[−0.400,-0.115]	*P* < 0.01	33.8%
Total effect	−0.775	0.059	[−0.890,-0.658]	*P* < 0.01	100%

## 4 Discussion

### 4.1 Self-efficacy, future time perspective, and occupational burnout levels among clinical nurses

The results of this study indicate that the total score for self-efficacy among clinical nurses is (25.97 ± 6.20), which is considered moderate and similar to the findings of Ding et al. (40–43). This may be due to the fact that nurses aged 20–30 years old constitute 65% of the study subjects, and their relatively limited work experience could contribute to a lack of the necessary confidence in nursing tasks. The total score for future time perspective is (43.46 ± 10.29), with a scoring rate of 67.90%, considered a moderate level and in line with the research by Mao et al. (44, 45). This could be attributed to the relative job stability of nurses, who may not feel pressured or urgent in their lives, resulting in a lack of a clear purposeful consciousness toward the future. The total score for occupational burnout is (49.15 ± 16.35), also at a moderate level, echoing the findings of Zhang et al. (46–48). Nurses may experience burnout due to the high workload, frequent night shifts, and irregular work-rest schedules in an environment where resources are limited and responsibilities are increasingly demanding, leading to an imbalance between providing quality nursing services and coping with significant stress (49–51). These findings suggest that nurse managers should make reasonable work arrangements for clinical nurses based on the actual situation.

### 4.2 Correlation between nurses’ self-efficacy, future time perspective and occupational burnout

This study reveals that there is a negative correlation between clinical nurses’ self-efficacy and occupational burnout, which is consistent with the findings of Zhang et al. (52, 53). Nurses with high self-efficacy tend to adopt an optimistic attitude when facing difficulties, are willing to choose challenging tasks, and when facing failure, they analyze the reasons and continually learn from their experiences, thus experiencing a lower level of occupational burnout (54, 55). Nurses with low self-efficacy often doubt their abilities when confronted with complex clinical problems, exaggerate issues, and this mindset is not conducive to resolving clinical problems or improving their skills, leading to a loss of enthusiasm for work and an increased susceptibility to burnout (56, 57). Therefore, hospital administrators should recognize the impact of psychological factors such as self-efficacy on occupational burnout. There should be a focus on nurses’ psychological well-being, with timely psychological counseling to help nurses address psychological issues, fostering confidence in their nursing work, and thereby reducing the degree of burnout. Furthermore, the study shows a positive correlation between self-efficacy and future time perspective, aligning with the research findings of Wang et al. (58). The development of an individual’s self-efficacy is related to the accumulation of past experiences of success or failure and is an important influencing factor of future time perspective. Therefore, nurses with high self-efficacy actively confront the present and future, are full of hope and anticipation, have a clear understanding of the future, and set definitive goals and directions, committing actions to achieve them. Additionally, future time perspective has a negative correlation with occupational burnout, which is in agreement with the research by Detaille et al. (59, 60). As a psychological variable, a positive future time perspective can help individuals set personal goals and implement plans effectively. Goal-oriented individuals have persistent and stable motivation and a proper understanding of their objectives (61–63). They take an interest in their work and invest more energy, which directly improves occupational burnout. Nursing managers should value the establishment of nurses’ personal goals, actively conduct training related to professional objectives, and encourage nurses to set short-term, medium-term, and long-term goals based on their actual situations. By striving to achieve these work goals, nurses can improve their future time perspective, thereby reducing the level of occupational burnout (64).

### 4.3 The mediating role of future time perspective between self-efficacy and occupational burnout

This study demonstrates that future time perspective plays a mediating role between clinical nurses’ self-efficacy and occupational burnout, with the mediation effect accounting for 33.8% of the total effect. This means that self-efficacy can influence the level of occupational burnout in clinical nurses through their future time perspective. Nurses with a high level of self-efficacy have stronger judgment in their actions and decisions, stronger self-confidence in handling the pressures and challenges of clinical work and life, and a clearer understanding of the future (65–69). They set clear goals and directions, are full of hope for the future, and face the present and future with an optimistic attitude. This, in turn, helps them actively deal with the adverse effects of occupational burnout and reduce its level (43, 70–73).

Nursing management should take steps to motivate nurses to enhance their self-efficacy, such as through organizing career planning for clinical nurses and providing training in the latest clinical skills to adapt to the work environment. Such measures can help nurses face various clinical challenges, boost their confidence, foster more positive expectations for the future, and make correct plans. By fully leveraging the core role of future time perspective, nurses can better realize their potential, thus reducing the level of occupational burnout, improving the overall quality of nursing services, maintaining the stability of the nursing team, and promoting the development of the nursing profession.

## 5 Strengths and limitations

This study is cross-sectional and was limited by resources, such as manpower, materials, and time, and did not implement intervention measures on clinical nurses. The research method was singular; future research could employ longitudinal surveys or qualitative studies. Additionally, the subjects of this study were from tertiary hospitals in Yibin City, and the results are limited by regional factors. Future research could involve multi-province, large-scale samples.

## 6 Conclusion

This study has demonstrated a significant interaction between clinical nurses’ self-efficacy, future time perspective, and occupational burnout. Future time perspective acts as a mediator between self-efficacy and occupational burnout, which is of great significance for in-depth research into the relationship between clinical nurses’ self-efficacy and occupational burnout. It suggests that nursing managers should recognize the important role of self-efficacy in reducing nurses’ occupational burnout. By enhancing comprehensive capabilities through further education and training, and by experiencing success, nurses can strengthen their self-efficacy. Intensifying professional training for nurses and continuously improving their professional qualifications and work skills can lead to a hopeful outlook for the future, thus reducing the level of occupational burnout and stabilizing the nursing workforce.

## Data availability statement

The original contributions presented in the study are included in the article/Supplementary material, further inquiries can be directed to the corresponding authors.

## Ethics statement

The studies involving humans were approved by the Jinzhou Medical University Ethics Committee (JZMULL2023152). The studies were conducted in accordance with the local legislation and institutional requirements. The participants provided their written informed consent to participate in this study.

## Author contributions

DX: Writing – original draft, Writing – review & editing. XZhu: Project administration, Formal analysis, Writing – review & editing. XZha: Data curation, Writing – review & editing. ZJ: Funding acquisition, Writing – review & editing. LT: Supervision, Validation, Writing – review & editing. XW: Writing – review & editing, Supervision, Validation.
